# Associations Between DCD Traits, Perceived Difficulties Related to ADHD, ASD, and Reading and Writing Support Needs Among Students in Higher Education: A Pilot Study

**DOI:** 10.3390/brainsci14111083

**Published:** 2024-10-29

**Authors:** Masanori Yasunaga, Ryutaro Higuchi, Keita Kusunoki, Chinatsu Mori, Naoto Mochizuki

**Affiliations:** 1 Health and Counseling Center, Osaka University, Toyonaka 560-0043, Japan; 2 Student Support Center, Bukkyo University, Kyoto 603-8301, Japan; 3 Bureau of Human Empowerment, University of Tsukuba, Tsukuba 305-8572, Japan

**Keywords:** higher education institutions, developmental coordination disorder, neurodevelopmental coordination disorder

## Abstract

**Background/Objectives:** Since the Act for Eliminating Discrimination against Persons with Disabilities was enacted in 2016, the number of students with disabilities in higher education institutions has increased significantly, creating a greater need for support. Developmental Coordination Disorder (DCD) often coexists with other neurodevelopmental disorders such as Autism Spectrum Disorder (ASD), Attention Deficit Hyperactivity Disorder (ADHD), and reading and writing difficulties; yet, awareness of DCD remains low. This study aimed to investigate the prevalence of DCD traits among Japanese higher education students and their relationship with ADHD, ASD, and reading and writing support needs **Methods:** A pilot study was conducted with 77 university students (mean age: 19.17 ± 0.68) in the Kansai region, using the Adolescents and Adults Coordination Questionnaire (AAC-Q) to screen for DCD traits, along with a questionnaire assessing difficulties related to ADHD and ASD, and a survey on reading and writing support needs. **Results:** The study found that 12.9% of students scored more than 1 standard deviation above the mean on the AAC-Q. Students in the DCD trait group (*n* = 10) reported greater difficulties related to ADHD and ASD than those in the non-DCD group (*n* = 67). Correlation analysis revealed a moderate-to-strong association between AAC-Q scores and difficulties related to ADHD and ASD among students in the DCD trait group. **Conclusions:** This study suggests the need to raise awareness about DCD traits and to provide comprehensive support for students with ADHD and ASD in Japanese higher education institutions.

## 1. Introduction

In the academic year 2019, the enrollment rate of students with disabilities in higher education institutions was approximately 20% in the U.S. [1] and 17.3% in the U.K. [2]. When considering the breakdown of disability types, in the U.S., 31% of students with disabilities had a learning disorder, 18% had Attention Deficit Hyperactivity Disorder (ADHD), and 15% had a psychiatric disability. In the U.K., 34% of students with disabilities had a specific learning disorder (SLD), 28% had a mental health condition, and 12% had two or more conditions. In contrast, following the implementation of the Act on the Elimination of Discrimination against Persons with Disabilities in Japan [3], a 2023 survey by the Japan Student Services Organization [4] reported that the total number of students with disabilities enrolled in higher education institutions was 58,141, representing 1.79% of the total student population. The most common disabilities among these students were psychiatric disabilities (33%), health impairments (26%), and developmental disorders (20%), indicating a 4.3-fold increase in the total number compared to the 2013 academic year.

In the U.S., the U.K., and Japan, tools such as the Autism Spectrum Quotient (AQ), Conners’ Adult ADHD Rating Scales (CAARS), and the Autism Diagnostic Observation Schedule, Second Edition (ADOS-2) are used to assess the characteristics of developmental disorders [5]. Additionally, in Japan, for developmental disorders such as Autism Spectrum Disorder (ASD) and ADHD, difficulty scales have been employed to explore the relationship between individual difficulties and maladjustment or secondary disabilities in university life [6], as well as to clarify the relationship between specific difficulties and behavioral problems, thereby promoting self-understanding [7].

Among developmental disorders, Developmental Coordination Disorder (DCD) has been reported to overlap with ADHD, ASD, SLD, and psychiatric disabilities [8], potentially affecting academic achievement.

DCD is defined as a condition in which the acquisition and execution of coordinated motor skills are significantly below the level expected for the individual’s age, without opportunities to learn and use those skills [9]. This lack of motor skills can interfere with daily activities, reduce participation frequency [10], and lead to mental health disorders [11]. It indicates that DCD is a lifelong chronic neurodevelopmental disorder. As educational and employment demands increase, many young people (aged 16–25) must cope with psychosocial challenges affecting mental health, life satisfaction, and self-esteem [12,13,14].

International reports include DCD and mobility issues as part of disability classifications in the U.S. [1] and physical impairments or mobility issues in the U.K. [2]. Furthermore, in the U.S., the Adult DCD/Dyspraxia Checklist is used to assess DCD characteristics [5].

However, in Japan, according to the classification by the Japan Student Services Organization [4], Developmental Coordination Disorder (DCD) is categorized as a physical function, and no independent survey specifically focusing on DCD has been conducted, leading to a possible low level of awareness of DCD among support staff. Additionally, complaints related to DCD are relatively few in higher education institutions, and there is a significant lack of research in Japan investigating the characteristics of DCD or examining the necessary accommodations for university students with DCD.

One possible reason for the under-recognition of DCD in Japan could be the lack of understanding within the medical, educational, and childcare sectors that DCD is a brain function disorder [15], as well as a lack of awareness that the interaction between physicality and the environment can affect brain function development. The prevalence of DCD is estimated to be 5–6% [9], and it has been reported that 50–70% of individuals continue to experience motor coordination difficulties into adulthood.

Additionally, recognizing DCD can be challenging because the symptoms of other neurodevelopmental disorders, such as ADHD, ASD, and SLD, are often more prominent or typical. Adults with DCD commonly experience not only motor difficulties but also issues with executive function, attention, anxiety, depression, and low self-esteem [8]. As a result, some university students may enter without a diagnosis of DCD [16]. Furthermore, it has been reported that there are very few professionals in higher education institutions who are adequately trained to diagnose or support adults with DCD [16]. Therefore, students with DCD traits likely exist in Japanese higher education institutions, and appropriate support from trained professionals is essential.

Hence, it is likely that students with DCD characteristics are present within Japan’s higher education institutions and need appropriate support. If support needs for DCD are not identified, it may negatively impact academic success, university life, employment, and social participation [17].

Therefore, it is essential for support staff at higher education institutions to understand the characteristics of DCD.

Although reports indicate that students feel a sense of clumsiness related to communication skills and a sense of adaptation [18], there has been inadequate research in Japan focusing on identifying DCD characteristics or investigating the accommodations required for students with DCD in universities.

This pilot study aimed to clarify the prevalence of DCD characteristics among university students in Japan and examine the relationship between these characteristics and difficulties associated with other developmental disorders, using the Adolescents and Adults Coordination Questionnaire (AAC-Q) [12] and questionnaires assessing difficulties related to developmental disorders [19,20,21]. The significance of this study lies in understanding how the relationship between DCD characteristics and ASD, ADHD, and SLD can help support staff adopt a broader perspective in recognizing DCD traits and effectively addressing the barriers to academic success faced by students with developmental disorders.

## 2. Materials and Methods

### 2.1. Participants

In this pilot study, we used a cross-sectional design to recruit undergraduate students aged 18–22 years in January 2024 from two Humanities and Social Sciences classes at a national university in the Kansai region of Japan.

**Figure 1:**
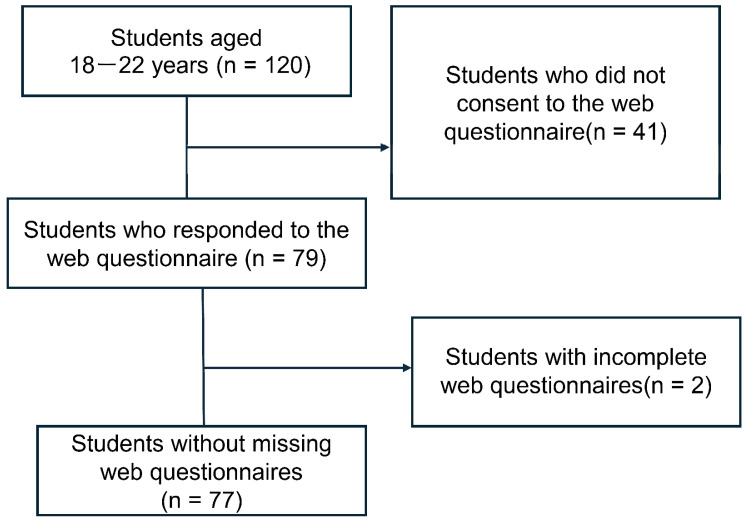
Flow chart of survey data collection.

Of the 120 students invited, 79 agreed to participate. After excluding two responses due to incomplete data, the final sample consisted of 77 participants (15 males, 61 females, and one other) (Figure 1). The mean age of the participants was 19.17 ± 0.68 years. The sample size was calculated using G*Power 3.1.9.7 [22] with a two-tailed test, an effect size of 0.3, a significance level of 0.05, and a power of 0.8. The minimum sample size required was calculated to be 80 participants.

### 2.2. Survey Procedure

The details of the study were explained to the students before class, and those who agreed to participate were included. Participants were instructed to read the explanation provided on the first screen of the online survey and to click the “Agree” button to proceed to the next page if they agreed to participate. Before the AAC-Q questions, participants received the instruction, “Read the questions and examples carefully, and if any example applies to you, respond based on that example”. Before the questions for the ASD Difficulty Scale, ADHD Difficulty Scale, LDSP7, and SCLD10, participants received the instruction, “This is a survey about the difficulties you experience in your daily life. Please respond on a 4-point scale according to the level of difficulty”.

### 2.3. Instruments

#### 2.3.1. The Adolescents and Adults Coordination Questionnaire

The AAC-Q [13] is a validated and standardized screening tool designed for individuals aged between 16 and 30. It is a self-report questionnaire consisting of 12 items rated on a 5-point Likert scale (1 = never, 5 = always). Higher total scores indicate more severe issues with developmental coordination. The questionnaire includes items related to daily activities (e.g., “I tend to be clumsy and often trip, drop items, or bump into objects”). The cutoff score of 27 or higher (above-1 SD) was used to identify individuals with DCD characteristics, referencing Tal-Saban [12].

The AAC-Q has excellent internal consistency, test-retest reliability, and discriminant validity [23]. Cronbach’s alpha for the AAC-Q was 0.88 [23]. As the AAC-Q has not been standardized in Japan, the developers provided a Japanese version with permission, and the content was reviewed and deemed appropriate for use by the first author and the co-authors.

#### 2.3.2. Developmental Disorders Difficulty Scales (Survey on Difficulties in University Life)

The Developmental Disorders Difficulty Scales are designed to assess the difficulties arising from students’ strengths and weaknesses and measure the extent to which students feel they are struggling with university life [19,20]. Specific scales have been developed for ADHD (hereafter referred to as the ADHD Difficulty Scale) and ASD (hereafter referred to as the ASD Difficulty Scale).

No cutoff points have been established, and the scales are structured to indicate students who fall within the top 5% percentile as well as to capture specific difficulties using subscales.

##### ADHD Difficulty Scale

The ADHD Difficulty Scale was developed by Takahashi [19,20] and uses a shortened version of the comprehensive difficulty scale, focusing on items strongly associated with ADHD tendencies. Participants rate their difficulties on a four-point scale (0 = not difficult at all, 3 = very difficult). The subscales included “Difficulty Maintaining Concentration” (1 item), “Inattention” (2 items), “Impulsivity” (2 items), “Lack of Planning Ability” (1 item), “Sleep Rhythm Disturbance” (1 item), and “Clumsiness” (2 items). Based on Takahashi [19,20], a total score of 1.4 or higher was used to identify participants with ADHD-related difficulties. Cronbach’s alpha for the ADHD Difficulty Scale was 0.88 [20].

##### ASD Difficulty Scale

The ASD Difficulty Scale, also developed by Takahashi [19,20], is a shortened version consisting of 13 items selected from the original 23-item comprehensive difficulty scale to reduce administration time and facilitate counseling. Participants rate their difficulties on a four-point scale (0 = not difficult at all, 3 = very difficult). The subscales included “Interpersonal Difficulties” (five items) and “Autism-Related Difficulties” (eight items). Based on Takahashi [19,20], a total score of 1.1 or higher was used to identify participants with ASD-related difficulties. Cronbach’s alpha for the ASD Difficulty Scale was 0.88 for the total score, 0.80 for the Autism-Related Difficulties subscale, and 0.84 for the Interpersonal Difficulties subscale [20].

#### 2.3.3. Reading and Writing Support Needs Scale

The Reading and Writing Support Needs Scale [21] (RaWSN) is designed to assess the difficulties and challenges related to reading and writing among university students. It also evaluates the underlying factors contributing to these challenges. The scale includes items that reflect the difficulties commonly experienced by individuals with SLD or dyslexia. Higher scores on this scale indicate a higher likelihood of an underlying SLD.

The RaWSN consists of 93 items in total: 44 items related to difficulties encountered in academic activities during university and 49 items related to difficulties experienced during elementary school. Various shortened versions of the scale are available for different purposes. This study used the Learning Difficulty Scale for Postsecondary Students (LDSP7) [21], which is suitable for screening students in need of support, and the Scale for Childhood Learning Difficulties (SCLD10) [21]. The LDSP7 includes seven items, and the SCLD10 includes ten items, both evaluated on a four-point scale (1 = Does not apply, 4 = Applies).

Higher scores on this scale suggest a higher likelihood of underlying SLD-related reading and writing difficulties. Based on Takahashi [21], a total score of 2.5 or higher was used to identify students with reading and writing difficulties. Cronbach’s alpha was 0.68 and 0.83 for the LDSP7 and the SCLD10, respectively [21].

### 2.4. Statistical Analysis

Statistical analyses were conducted using SPSS for Windows version 26 (IBM Corp., Armonk, NY, USA). As most measurements did not follow a normal distribution, nonparametric analyses were employed. The normality of the two groups (DCD characteristics present and absent) was assessed using the Shapiro–Wilk test. The reliability of each assessment (AAC-Q, ASD Difficulty Scale, ADHD Difficulty Scale, LDSP7, and SCLD10) was calculated using Cronbach’s alpha. Welch’s *t*-test was used to analyze differences in the total scores and subscales of the four assessments and age differences between the two groups. Gender differences were analyzed using the χ^2^ test.

Additionally, Spearman’s rank correlation coefficient was used to examine the associations between the AAC-Q and the ADHD/ASD Difficulty Scales, LDSP7, and SCLD10 for all participants and within the two groups. A significance level of *p* < 0.05 was set, and an alpha level of less than 0.05 was considered significant. The sample size for correlation analysis was determined using G*Power 3.1.9.7 [22]. This study interpreted effect sizes following Cohen’s (2013) guidelines. Specifically, Cohen’s [24] d values of 0.2, 0.5, and 0.8 were classified as small, medium, and large effect sizes, respectively. Additionally, power levels of 80% (0.80) or higher were deemed appropriate for determining the sample size.

### 2.5. Ethical Considerations

This study was approved by the Ethics Committee of the Osaka University Health and Counseling Center (HaCC) (Approval No. 10). At survey commencement, participants were informed that all data and personal information collected in this study would be statistically processed, protected by passwords, and not accessible to any third party not involved in the study.

Participants were also informed that all data would be deleted after data collection. Moreover, they were informed that the study results would be published in academic journals and related publications without identifying information and that their anonymity would be maintained.

## 3. Results

### 3.1. Overall Results

On the AAC-Q, five participants (9%) scored above 2 SD, and 10 participants (12.9%) scored above 1 SD. On the ADHD Difficulty Scale, two participants (2.6%) scored above 2 SD, and 12 participants (15.6%) scored above 1 SD. On the ASD Difficulty Scale, four participants (5.2%) scored above 2 SD, and 12 participants (15.6%) scored above 1 SD. None of the students scored above 2 SD or 1 SD on the LDSP7 and SCLD10. Students in the group with DCD traits (AAC-Q score of 27 or higher) exhibited higher levels of difficulty in all ADHD (total score, concentration, inattention, impulsivity, planning, and clumsiness) and ASD items (total score, autism-related, and interpersonal), except for ADHD-related sleep difficulties, compared to the group without DCD traits (Table 1).

Cronbach’s alpha coefficients for the four tests used in this study were as follows: AAC-Q, 0.90; ADHD Difficulty Scale, 0.92; ASD Difficulty Scale total, 0.88; Autism-related Difficulties, 0.81; Interpersonal Difficulties, 0.81; LDSP7, 0.77; SCLD10, 0.79.

### 3.2. Correlation Among AAC-Q, ADHD Difficulty Scale, ASD Difficulty Scale, LDSP7, and SCLD10

Correlation coefficients among the AAC-Q, ADHD Difficulty Scale, ASD Difficulty Scale, LDSP7, and SCLD10 were calculated. The AAC-Q total score moderately correlated with the ADHD total score (r = 0.65, *p* < 0.01), ADHD concentration (r = 0.52, *p* < 0.01), inattention (r = 0.59, *p* < 0.01), clumsiness (r = 0.57, *p* < 0.01), LDSP7 total score (r = 0.51, *p* < 0.01), and SCLD10 total score (r = 0.51, *p* < 0.01) (Table 2).

### 3.3. Correlation Between AAC-Q, ADHD Difficulty Scale, ASD Difficulty Scale, LDSP7, and SCLD10 in Groups with and Without DCD Traits

Correlation coefficients were calculated between the AAC-Q, ADHD Difficulty Scale, ASD Difficulty Scale, LDSP7, and SCLD10 for the two groups (with and without DCD traits). In the group with DCD traits, the AAC-Q total score was strongly correlated with the ADHD Difficulty Scale total score (r = 0.86, *p* < 0.01), clumsiness (r = 0.84, *p* < 0.01), concentration (r = 0.78, *p* < 0.01), and impulsivity (r = 0.73, *p* < 0.05) (Table 3). Additionally, a strong correlation was observed between the AAC-Q total score and the ASD total score (r = 0.72, *p* < 0.05) as well as autism-related difficulties (r = 0.72, *p* < 0.05) (Table 3).

Contrastingly, in the group without DCD traits, the AAC-Q total score showed a moderate correlation with the ADHD Difficulty Scale total score (r = 0.51, *p* < 0.01), inattention (r = 0.41, *p* < 0.01), and clumsiness (r = 0.44, *p* < 0.01). The AAC-Q total score showed a weak correlation with the ASD Troublesomeness Scale total score (r = 0.25, *p* > 0.05) and ASD autism (r = 0.28, *p* > 0.05).

## 4. Discussion

### 4.1. Prevalence of DCD Traits and Difficulties with ADHD, ASD, and SLD in Higher Education Institutions

This study suggests that some university students possess DCD traits. DCD is reported to persist into adulthood for 30–70% of cases, and its occurrence is not limited to school age [25]. This finding is consistent with those of previous studies. Furthermore, this indicates that students in higher education institutions in late adolescence may have DCD traits and require appropriate accommodation.

In this study, two students (2.6%) scored above 2 SD on the ADHD Difficulty Scale, and four students (5.2%) scored above 2 SD on the ASD Difficulty Scale. The proportion of students who scored below the cutoff values on these two difficulty scales was similar to the findings of Harada [6] and Shinoda [7]. No student scored above the cutoff values on the LDSP7 or SCLD10 scales. This may be because the study targeted students who had passed university entrance exams, suggesting that those with severe reading and writing difficulties or significant limitations in academic activities in the university environment may have been unable to participate. Hence, the 77 participants in this study may have had a low likelihood of reading and writing difficulties and, although somewhat atypical, were similar to the groups studied by Harada and Shinoda [6,7].

### 4.2. Association Between DCD Traits and ADHD, ASD, and SLD Difficulties in 77 Participants

Participants showed a moderate association between DCD traits and difficulties related to ADHD and ASD, indicating that stronger DCD traits were associated with greater difficulties with ADHD and ASD. Additionally, there was a moderate association between DCD traits and the LDSP7 and SCLD10 scores, suggesting that stronger DCD traits are linked to greater reading and writing difficulties. DCD often co-occurs with ADHD and ASD [8], and it is expected that individuals with DCD traits will experience difficulties related to ADHD and ASD. Internationally, students with DCD in higher education institutions often exhibit comorbid SLD, and accommodations are provided accordingly [26]. When comparing the number of students with different disabilities in Japan between 2013 [27] and 2023 [4], the number of students diagnosed with SLD increased by approximately 2.2 times (228 students). However, this was still lower than the number of students with ASD (4640 students) or ADHD (3421 students), and DCD is not included as a category in the Japan Student Services Organization [4].

According to Blank et al. [8], only about 15% of individuals with DCD do not have comorbid conditions, with many exhibiting comorbid ADHD [28], ASD [29,30], or psychiatric disorders, often co-occurring with SLD [31].

Considering these findings, students with ASD, ADHD, or SLD traits may also have DCD traits but prioritize accommodations related to communication, sensory issues, schedule management, reading, and writing, potentially overlooking their DCD traits or not recognizing their need for accommodation. Therefore, it is important for support staff to consider the possibility of co-occurring DCD traits and adopt a broad perspective while assessing difficulties for students with difficulties related to ASD, ADHD, or SLD. This approach could lead to a better understanding of student characteristics and help identify the social barriers to their academic activities.

### 4.3. Association Between AAC-Q and Developmental Disability Difficulty Scales in Groups with and Without DCD Traits

When comparing the group with DCD traits and the group without DCD traits, the group with DCD traits had higher difficulty scores on most items of the ADHD and ASD difficulty scale. Additionally, there was a moderate-to-strong correlation between the AAC-Q and ADHD/ASD difficulties and between the ADHD total score and ASD total score in the group with DCD traits. These findings suggest that students with DCD traits have a greater chance of experiencing difficulties related to ADHD and ASD than those without DCD traits.

Internationally, the awareness and knowledge of DCD among support staff and students in higher education institutions is low [32]. Students with DCD receive less support for ICT and writing skills than students with dyslexia (a form of SLD) [33]. Similarly, in Japan, there are a few reports of students expressing concerns about the difficulties or accommodations related to DCD in higher education institutions. Three possible factors may contribute to this situation. The first factor is the lack of accommodation implementation, specifically targeting difficulties arising from DCD. According to the Japan Student Services Organization [4], accommodations for developmental disorders often include “distribution of accommodation request documents”, “substitution of instructional content, extension of assignment deadlines”, and more, but few directly address DCD. Koyama et al. [34] reported alternative accommodations for physical education and practical subjects, but these accommodations mainly targeted visual, auditory, and physical disabilities, not developmental disorders or DCD [35]. Takahashi et al. [5] reported that accommodations such as extended deadlines and exam times were provided to students with ASD, ADHD, and DCD traits who had writing or typing difficulties. However, these accommodations were insufficient. These findings suggest that support staff may lack sufficient knowledge and understanding of DCD traits and the necessary accommodation. In other countries, students with DCD receive less financial support for accommodation than students with dyslexia or those with dyslexia and DCD, leading to less accommodation being provided [33]. Therefore, it is essential for support staff in higher education institutions to be aware of difficulties related to developmental disorders, DCD traits, and appropriate accommodations. The second factor is that students with DCD-related difficulties can independently manage their course selections and environmental adjustments. Unlike the provision of reasonable accommodations for students with developmental disorders [36], students with DCD traits may choose courses that focus on sitting and listening to lectures (avoiding practical or physical activities involving fine motor skills). Forde [37] suggested that adults with DCD tend to avoid potentially distressing social situations, including school and work, in their daily lives. Furthermore, universities tend to avoid courses that require high physical abilities, such as physical sciences and health courses [16]. This suggests that students with DCD traits independently choose avoidance strategies for activities that they find challenging. The third factor is that students with developmental or psychiatric difficulties may be unaware of DCD traits. Previous studies have reported cases in which individuals were diagnosed with DCD after entering graduate school [38] or after receiving psychiatric care after high school [39]. In adulthood, DCD symptoms are usually accompanied by secondary psychiatric disorders (mood disorders and anxiety) [40,41], and co-occurrence with other disorders and disabilities is frequently observed [40,41,42], leading to more complex symptoms.

Based on these findings, adults with DCD are more likely to experience difficulties related to depression, anxiety, and interpersonal relationships rather than motor issues, making DCD-related challenges harder to recognize than in childhood. International reports indicate that students with DCD struggle not only with handwriting and PC skills but also with organization and time management due to weaker executive functions [12]. These difficulties overlap with ADHD traits. Additionally, students with DCD are reportedly less likely to receive ICT and writing support than those with dyslexia [33], suggesting that they may be coping with challenges through self-effort alone. Therefore, it is necessary to recognize DCD-related difficulties early through dialogue between the student and support personnel.

These findings indicate a lack of understanding among support personnel regarding DCD characteristics, a tendency for students with DCD traits to avoid courses involving practical skills at enrollment, and that many students are unaware of their DCD traits, resulting in limited consultation or support. Thus, it is necessary to conduct DCD screening during the intake process and to provide tailored support measures for students with developmental challenges. In this study, approximately 10% of students were found to have DCD traits. There was a strong correlation between students with DCD traits and significantly higher difficulties related to ADHD and ASD compared to those without DCD traits. Therefore, DCD screening and tailored support services are essential for students with developmental challenges receiving accommodations in higher education.

### 4.4. Limitations

This study had several limitations. First, the study was limited to University A (a national university) and primarily included first-year undergraduate students from Humanities and Social Sciences classes, resulting in a restricted sample size. Additionally, the overall sample and the DCD characteristics group were predominantly female.

Second, the assessment of DCD traits, ADHD, ASD difficulties, and literacy support needs relied on self-reported questionnaires. Additionally, the cutoff values for the ACC-Q were based on overseas data, making it necessary to reconsider appropriate cutoff values for Japan. These factors limit the generalizability of the findings. Third, due to the small sample size, we were unable to conduct factor analysis for construct validity or criterion-related validity, leaving the AAC-Q validity based solely on subjective face validity. Fourth, in the non-DCD group, the standard deviation for the ADHD difficulties scale exceeded the mean, and there was variability in the confidence intervals. As a result, the interpretation of the correlation results may have been limited.

Fifth, the “I struggle with organizing and tidying up” item in the ADHD difficulties section of the Developmental Disorders Difficulty Scales questionnaire was unintentionally omitted due to a settings error. Lastly, factors such as students’ academic levels were not accounted for in the interpretation of the results.

### 4.5. Future Research

In future research, the construct validity and criterion-related validity of the AAC-Q should first be evaluated and confirmed. Then, a larger, more representative sample should be secured by conducting a nationwide survey across higher education institutions, considering factors such as academic department, major, academic year, and gender ratio, and incorporating objective assessments of each trait.

A stronger association between DCD traits and ADHD/ASD difficulties will further emphasize the importance of screening for DCD traits in students with developmental disorders. Additionally, to identify social barriers specific to DCD traits, interviews should be conducted with the aforementioned students to explore their unique academic activities and desired accommodations related to DCD traits.

## 5. Conclusions

This pilot study was conducted on a limited group of students from a Japanese university. Although the study was small in scale, it found that a certain number of students in higher education exhibit DCD traits, which are associated with challenges related to other developmental disorders. Therefore, considering DCD traits when providing support to students with developmental disorders may be crucial for offering more effective assistance. Future research should expand the sample to include universities from various regions in Japan, considering diverse attributes to explore these findings further.

## Figures and Tables

**Table 1:** Comparison of assessment results between groups with and without DCD traits.

Outcome Measure	Group with DCD Traits		Group Without DCD Traits			
	(*n* = 10)		(*n* = 67)			
	Mean (SD)	Median	Mean (SD)	Median	*p*-Value	Effect Size
		(25th–75th Percentile)		(25th–75th Percentile)		
Sex	1 male, 9 females		14 males, 52 females, 1 other		0.001 ^†^	0.14
Age	19.2 (0.79)	19 (18.75–20)	19.16 (0.67)	19 (19–19)	0.894 ^‡^	0.02
AAC-Q						
Total score	37.1 (7.14)	35 (31.25–43.25)	17.75 (4.49)	18 (14–22)	0.000 ^‡^	0.93
ADHD						
Total score	15.2 (6.60)	14.5 (9.50–18.75)	5.24 (4.74)	4 (1–8)	0.000 ^‡^	0.56
Concentration	2.2 (0.92)	2 (1.75–3.00)	0.78 (0.78)	1 (0–1)	0.000 ^‡^	0.52
Inattention	4.0 (1.56)	4 (2.75–6.00)	1.12 (1.67)	0 (0–2)	0.000 ^‡^	0.51
Impulsivity	2.1 (1.60)	1.5 (1.0–3.0)	0.61 (1.01)	0 (0–1)	0.000 ^‡^	0.42
Sleep	1.4 (1.26)	1 (0–3.0)	0.79 (0.93)	0 (0–1)	0.070 ^‡^	0.21
Planning	1.7 (1.42)	1.5 (0.75–2.50)	0.60 (0.89)	0 (0–1)	0.001 ^‡^	0.36
Clumsiness	3.8 (1.75)	4 (2.0–4.25)	1.34 (1.40)	0 (0–2)	0.000 ^‡^	0.5
ASD						
Total score	16.3 (7.33)	16.5 (10–21.25)	5.78 (5.29)	4 (2–8)	0.000 ^‡^	0.54
Autism	10.6 (4.17)	11 (6.0-14.25)	3.33 (3.19)	3 (1–5)	0.000 ^‡^	0.6
Interpersonal	5.7 (3.47)	6 (3.25–7.75)	2.45 (2.76)	1 (0–4)	0.001 ^‡^	0.36
SLD						
LDSPt7 total score	12.1 (3.51)	13 (8.75–14)	7.96 (1.55)	7 (7–9)	0.005 ^‡^	0.6
SCLD10 total score	15 (2.98)	15 (11.75–18)	11.21 (2.54)	10 (10–11)	0.000 ^‡^	0.45

AAC-Q: The Adolescents and Adults Coordination Questionnaire; ADHD: ADHD Distress Questionnaire (Short Version); ASD: Distress Questionnaire (Short Version); LDSPt7: LDSPt7 Questionnaire; SCLD10: SCLD10 Questionnaire; ^†^ chi square test; ^‡^ Welch test.

**Table 2:** Relationship between AAC-Q and ADHD, ASD, SLD (*n* = 77).

	1	2	3	4	5	6	7	8	9	10	11	12	13
1. AAC-Q-TS	―												
2. ADHD-TS	0.650 **	―											
	[0.49,0.77]												
3. ADHD-CO	0.515 **	0.781 **	―										
	[0.33,0.67]	[0.67,0.86]											
4. ADHD-INA	0.588 **	0.773 **	0.593 **	―									
	[0.41,0.72]	[0.66,0.85]	[0.42,0.72]										
5. ADHD-IM	0.462 **	0.704 **	0.606 **	0.514 **	―								
	[0.26,63]	[0.57,0.80]	[0.44,0.73]	[0.32,0.67]									
6. ADHD-SL	0.318 **	0.609 **	0.352 **	0.263 *	0.299 **	―							
	[0.09,0.51]	[0.44,0.74]	[13,0.54]	[0.04,0.47]	[0.07,0.50]								
7. ADHD-PL	0.446 **	0.739 **	0.472 **	0.597 **	0.457 **	0.437 **	―						
	[0.24,0.61]	[0.61,0.83]	[0.27,0.63]	[0.43,0.73]	[0.25,0.62]	[0.23,0.61]							
8. ADHD-CL	0.572 **	0.812 **	0.584 **	0.537 **	0.499 **	0.409 **	0.595 **	―					
	[0.39,0.71]	[0.72,0.88]	[0.41,0.72]	[0.35,0.68]	[0.30,0.65]	[0.20,0.58]	[0.42,0.73]						
9. ASD-TS	0.443 **	0.501 **	0.408 **	0.279 *	0.556 **	0.316 **	0.195	0.545 **	―				
	[0.24,0.61]	[0.31,0.66]	[0.20,0.58]	[0.05,0.48]	[0.37,0.70]	[0.09,0.51]	[−0.04,0.41]	[0.36,0.69]					
10. ASD-AU	0.481 **	0.625 **	0.473 **	0.379 **	0.610 **	0.411 **	0.337 **	0.588 **	0.903 **	―			
	[0.28,0.64]	[0.46,0.75]	[0.27,0.63]	[0.16,0.56]	[0.44,0.74]	[0.20,0.59]	[0.12,0.53]	[0.41,0.72]	[0.85,0.94]				
11. ASD-INT	0.283 *	0.286 *	0.298 **	0.126	0.416 **	0.154	0.045	0.395 **	0.876 **	0.614 **	―		
	[0.06,0.48]	[0.06,0.48]	[0.07,0.49]	[−0.11,0.35]	[0.20,0.59]	[−0.08,0.37]	[−0.19,0.27]	[0.18,0.57]	[0.81,0.92]	[0.45,0.74]			
12. LDSPt7-TS	0.508 **	0.394 **	0.336 **	0.308 **	0.401 **	0.054	0.141	0.527 **	0.424 **	0.434 **	0.331 **	―	
	[0.31,0.66]	[0.18,0.57]	[0.11,0.53]	[0.08,0.50]	[0.19,0.58]	[−0.18,0.28]	[−0.09,0.36]	[0.34,0.68]	[0.21,0.60]	[0.23,0.60]	[0.11,0.52]		
13. SCLD10-TS	0.514 **	0.392 **	0.312 **	0.414 **	0.323 **	0.035	0.240 *	0.494 **	0.364 **	0.341 **	0.346 **	0.659 **	―
	[0.32,0.67]	[0.18,0.57]	[0.09,0.51]	[0.20,0.59]	[0.10,0.51]	[−0.20,0.26]	[0.01,0.45]	[0.30,0.65]	[0.15,0.55]	[0.12,0.53]	[0.13,0.53]	[0.51,0.77]	

* *p* < 0.05. ** *p* < 0.01. [ ] indicate the 95% confidence interval. AAC-Q: The Adolescents and Adults Coordination Questionnaire; ADHD: ADHD Distress Questionnaire (Short Version); ASD: Distress Questionnaire (Short Version); LDSPt7: SLD LDSPt7 Questionnaire; SCLD10: SLD SCLD10 Questionnaire; TS: total score; CO: concentration; INA: inattention; IM: impulsivity; SL: sleep; PL: planning; CL: clumsiness; AU: autism; INT: interpersonal.

**Table 3:** Relationship between AAC-Q and ADHD, ASD, and SLD (group with DCD traits, *n* = 10).

	1	2	3	4	5	6	7	8	9	10	11	12	13
1. AAC-Q-TS	―												
2. ADHD-TS	0.857 **	―											
	[0.48,0.97]												
3. ADHD-CO	0.784 *	0.888 **	―										
	[0.28,0.95]	[0.57,0.97]											
4. ADHD-INA	0.505	0.728 *	0.470	―									
	[−0.20,0.87]	[0.16,0.93]	[−0.25,0.85]										
5. ADHD-IM	0.728 *	0.744 *	0.755 *	0.416	―								
	[0.16,0.93]	[0.20,0.94]	[0.22,0.94]	[−0.31,0.84]									
6. ADHD-SL	0.208	0.499	0.615	0.195	0.081	―							
	[−0.50,0.75]	[−0.21,0.86]	[−0.05,0.90]	[−0.51,0.74]	[−0.59,0.69]								
7. ADHD-PL	0.643 *	0.768 **	0.668 *	0.268	0.598	0.532	―						
	[0.00,0.91]	[0.25,0.94]	[0.04,0.92]	[−0.45,0.78]	[−0.07,0.90]	[−0.17,0.88]							
8. ADHD-CL	0.838 **	0.902 **	0.823 **	0.695 *	0.655 *	0.310	0.577	―					
	[0.42,0.96]	[0.62,0.98]	[0.38,0.96]	[0.09,0.92]	[0.02,0.91]	[−0.42,0.79]	[−0.10,0.89]						
9. ASD-TS	0.723 *	0.710 *	0.660 *	0.527	0.712 *	0.006	0.332	0.717 *	―				
	[0.15,0.93]	[0.12,0.93]	[0.03,0.91]	[−0.18,0.87]	[0.13,0.93]	[−0.64,0.65]	[−0.39,0.80]	[0.14,0.93]					
10. ASD-AU	0.717 *	0.704 *	0.641 *	0.577	0.681 *	0.032	0.288	0.658 *	0.978 **	―			
	[0.14,0.93]	[0.11,0.93]	[0.00,0.91]	[−0.10,0.89]	[0.07,0.92]	[−0.62,0.66]	[−0.43,0.79]	[0.03,0.91]	[0.90,10.0]				
11. ASD-INT	0.606	0.572	0.567	0.330	0.760 *	−0.146	0.277	0.643 *	0.905 **	0.831 **	―		
	[0.06,0.90]	[−0.11,0.89]	[−0.12,0.89]	[−0.40,0.80]	[0.23,0.94]	[−0.72,0.55]	[−0.45,0.78]	[0.00,0.91]	[0.63,0.98]	[0.41,0.96]			
12. LDSPt7-TS	0.148	0.265	0.208	−0.378	0.251	−0.729 *	−0.279	−0.155	0.222	0.189	0.433	―	
	[−0.55,0.72]	[−0.78,0.45]	[−0.75,0.50]	[−0.82,0.35]	[−0.47,0.77]	[−0.93,0.16]	[−0.78,0.44]	[−0.73,0.54]	[−0.49,0.76]	[−0.52,0.74]	[−0.29,0.84]		
13. SCLD10-TS	0.270	0.298	0.233	0.003	−0.200	−0.140	−0.363	−0.401	−0.138	−0.025	−0.403	−0.127	―
	[−0.78,0.45]	[−0.79,0.43]	[−0.76,0.48]	[−0.64,0.64]	[−0.75,0.51]	[−0.72,0.55]	[−0.82,0.36]	[−0.83,0.33]	[−0.72,0.55]	[−0.66,0.63]	[−0.83,0.32]	[−0.71,0.56]	

* *p* < 0.05. ** *p* < 0.01. [ ] indicate the 95% confidence interval. AAC-Q: The Adolescents and Adults. Coordination Questionnaire; ADHD: ADHD Distress Questionnaire (Short Version); ASD: Distress Questionnaire (Short Version); LDSPt7: SLD LDSPt7 Questionnaire; SCLD10: SLD SCLD10. Questionnaire; TS: total score; CO: concentration; INA: inattention; IM: impulsivity; SL: sleep; PL: planning; CL: clumsiness; AU: autism; INT: interpersonal.

## Data Availability

The study data can be accessed via the following website: https://doi.org/10.6084/m9.figshare.26525971 accessed on 31 August 2024.
